# Research and Application of Terahertz Response Mechanism of Few-Layer Borophene

**DOI:** 10.3390/nano12152702

**Published:** 2022-08-05

**Authors:** Zhixun Zhang, Mingyang Yang, Yibo Zhang, Ming Zhou

**Affiliations:** 1 State Key Laboratory of Tribology, School of Mechanical Engineering, Tsinghua University, Beijing 100084, China; 2 Key Laboratory for Advanced Materials Processing Technology, Ministry of Education, Tsinghua University, Beijing 100084, China

**Keywords:** borophene, terahertz, application, mechanism, first principles

## Abstract

The terahertz stealth and shielding performance of a new type of two-dimensional material, borophene, has been studied theoretically and experimentally. Studies have shown that borophene materials have good terahertz stealth and shielding properties. First-principles calculations show that compared with single-layer borophene, few-layer borophene has good terahertz stealth and shielding performance in the range of 0.1~2.7 THz. In the range of 2~4 layers, the terahertz stealth and shielding performance of few-layer borophene increases with the increase of the number of layers. The finite element simulation calculation results also confirmed this point. Using the few-layer borophene prepared by our research group as a raw material, a PDMS composite was prepared to verify the terahertz stealth and shielding performance of the few-layer borophene. In the ultra-wide frequency range of 0.1~2.7 THz, the electromagnetic shielding effectiveness (EMI SE) of the PDMS material mixed with few-layer borophene can reach 50 dB, and the reflection loss (RL) can reach 35 dB. With the concentration of few-layer borophene increasing, the terahertz stealth and shielding effectiveness of the material is enhanced. In addition, the simultaneous mixing of few-layer borophene and few-layer graphene will make the material exhibit better terahertz stealth and shielding performance compared with mixing separately.

## 1. Introduction

Terahertz wave refers to the electromagnetic wave band in the range of 0.1~10 THz, and its wavelength range is 0.03~3 mm [1]. Since the wavelength of terahertz is located between microwave and infrared, it has the characteristics of both, occupying a very important position in modern science and technology, and has unlimited development prospects in the fields of national defense, meteorology, and medical treatment [2,3]. Terahertz wave has the characteristics of a high degree of confidentiality, good directionality, strong penetrating ability, large information capacity, and high security performance, which makes it have broad application prospects in the detection field [4,5,6]. One prominent application of terahertz detection technology is terahertz radar detection technology. Due to its high penetration, directionality, and high confidentiality, terahertz radar occupies an important position in the military industry. In recent years, scientists from many countries have carried out research in the field of terahertz radar detection and have made good progress [7,8,9]. With the existing stealth methods seldom effective for terahertz waves, the research and development of terahertz radars have great significance for anti-stealth detection, and many countries have invested a lot of funds in the research and application of terahertz radars.

With the development of terahertz detection technology and detection radars, the application of terahertz technology in the military field is continuously expanding, causing major changes in the existing information warfare model. To take the lead in the military game, it is very important for military equipment to achieve shielding and stealth in the terahertz frequency band. The first to master related technologies will have an advantage in electromagnetic protection and information confidentiality in military confrontation. Therefore, it is very important to develop new terahertz stealth and shielding materials. Generally, people call materials with high absorptivity and low reflectivity in the terahertz band terahertz stealth materials. The stealth materials on the existing weapons and military equipment are mainly designed for the currently mainstream gigahertz detection radars, which can effectively reduce the scattering cross-sectional area of gigahertz radars, thereby achieving the absorption and attenuation of gigahertz waves [10]. However, the stealth material has no obvious effect on the stealth of terahertz. The wavelength of terahertz waves is shorter than that of gigahertz waves. Therefore, materials with low reflectivity to terahertz waves have a different chemical structure than that of gigahertz stealth materials, and terahertz stealth materials have higher requirements for surface structure. The research on terahertz stealth materials is now mainly based on dielectric loss materials to realize the dielectric absorption loss of terahertz waves. Common terahertz stealth materials can be divided into two categories: metamaterials and carbon materials.

Metamaterials are materials whose main structure is absorption. By designing the micro-nano-structure of the material, the resonance absorption of terahertz waves is achieved. Metamaterials can achieve effective absorption of specific frequency waves through simple structural design [11,12]. However, it is difficult to achieve full absorption of broadband waves, and the incident angle and polarization direction of terahertz waves will also affect its absorption effect. Carbon materials are also known as carbon-based absorber materials. It is a material that achieves terahertz stealth based on the intrinsic absorption of the material. Compared with metamaterials, carbon materials have rich structures, modulated electromagnetic properties, and easy advantages of preparation [13,14]. However, due to the poor dispersion of carbon materials in composite materials, it is difficult to form an efficient network structure and the interface mismatch between the material surface and the free space surface results in a large number of reflection restrictions at the interface, which greatly affects the stealth performance of carbon materials. Therefore, the improvement of the stealth performance of carbon materials requires a breakthrough in its structure and electromagnetic properties. Terahertz shielding materials refer to materials with low transmittance to terahertz waves. Carbon materials represented by graphene also exhibit excellent properties in terahertz shielding [15].

Compared with traditional metal-based materials, carbon materials have the characteristics of light weight and high efficiency [14]. Scientists have put forward the requirements of ultra-wideband, high absorption rate, and ultra-thinness for terahertz stealth and shielding materials. Boron is the element that comes before carbon in the periodic table. The two-dimensional material borophene and quantum dots formed by it have shown excellent optical, mechanical, and electronic properties in the calculations of scientists. The terahertz shielding and stealth performance is very meaningful. In addition, compared with graphene, the ^10^B element in borophene and boron quantum dots is the existing material that efficiently absorbs thermal neutrons. If borophene and other materials are made into terahertz stealth and shielding materials, it will play an important role in national defense and military. Recently, our research group has initially explored the terahertz response performance of few-layer borophene in the process of studying the stability and properties, and found that it has excellent terahertz stealth and shielding performance. However, the mechanism of terahertz stealth and shielding of few-layer borophene has not been systematically studied and analyzed.

In this article, we used the first principles to calculate the dielectric response function and other parameters of the single-layer and few-layer borophene, and analyzed and studied the electronic properties of the single-layer and the few-layer borophene. Then, calculation and research have obtained the terahertz absorption and reflection properties of single-layer and few-layer borophene by using the calculated optical parameters. The terahertz stealth and shielding performance of single-layer and few-layer borophene have been analyzed as well. It was found that due to its high optical transparency, the single-layer borophene transmits almost all terahertz waves, and does not have terahertz stealth and shielding properties. With the increase of the number of layers, the electromagnetic shielding effectiveness in the range of 0.1 to 2.7 THz gradually increased. The maximum electromagnetic shielding effectiveness (at 2.7 THz) changed from that of the two-layer borophene, to 35.65–42.98 dB of three-layer borophene, and then 45.24 dB of four-layer borophene. The reflection loss gradually decreased with the increase of the number of layers, but the decreasing trend gradually slowed down, and the reflection loss value was always greater than 10 dB. In addition, the finite element method was used to analyze and calculate the terahertz absorption and reflection properties of single-layer and few-layer borophene, and the results were basically consistent with the first-principles calculation results. On the other hand, using the terahertz wave-transmitting material, PDMS, as the substrate, a composite material was prepared by mixing few-layer borophene, and the terahertz time-domain spectroscopy system was used to experimentally verify the terahertz response performance of the composite material. The results indicated that its terahertz shielding performance can reach 50 dB, and the reflection loss can reach 35 dB. It was also found that as the mixing concentration of borophene increased, the electromagnetic shielding effectiveness of the composite material increased. Finally, the effect of mixing graphene on the terahertz response performance of PDMS composites mixed with few-layer borophene was studied, and it was found that the simultaneous mixing of graphene and few-layer borophene will make the composites exhibit better terahertz shielding performance.

## 2. Methods

### 2.1. Experimental Methods

An appropriate amount of the few-layer borophene powder prepared after purification and drying was placed in an agate mortar and ground for 10 min to ensure that the agglomerates caused by the drying process were all crushed and the few-layer borophene powder had a fine and uniform particle size. Then, 1 g of ground few-layer borophene powder, 3 g of PDMS base solution, and 6 g of cyclohexane as a diluent were weighed [16]. The mixture was stirred via magnetic stirring at 200 r/min for 10 min to make the few-layer borophene uniformly disperse in the PDMS glue. Then, 0.3 g of PDMS curing agent was added and stirred again for 1 min. It was placed in a freeze-dryer after mixing uniformly, and the vacuum was turned on for 10 min. The purpose is to use the vacuum function of the dryer to remove the bubbles generated by stirring in the mixed glue. The removed mixed glue was poured into a prepared 50 × 50 mm mold and put in an oven at a low temperature of 60 °C for 8 h to solidify the PDMS film formation.

### 2.2. First-Principles Simulation

The calculation model was built using Material Studio software, Accelrys, San Diego, CA, USA. The thickness of the vacuum layer of the model was set to 15 Å. The simulation calculation used the VASP software package, VASP Software GmbH, Vienna, Austria (VASP 5.3.3) for calculation. In the calculation process, PAW (Projector-Augmented Wave) was selected as the pseudopotential, and for Perdew–Burke–Ernzerhof (PBE), the cutoff energy was set to 400 eV. The surface Brillouin zone was divided by 3 × 3 × 1 k-point (Monkhorst–Pack). All structures fully relaxed in the calculation process until the force on each atom was less than 0.05 eV/Å, and for considering the van der Waals interactions, the DFT-D2 method of Grimme was used.

It should be noted that according to previous work, for metals such as Cu and Ag, when calculating the dielectric function, it is necessary to consider the effects of inter-band and intra-band transitions. For non-metallic materials such as semiconductors, when calculating the dielectric function, only the influence of the inter-band transition needs to be considered, and the result can be calculated with sufficient accuracy [17]. The properties of the few-layer borophene studied in the paper show semiconductor properties [18], and the boron itself is also a non-metallic material. Therefore, only the contribution of the inter-band was considered when calculating the dielectric function in this article, ensuring sufficient accuracy and saving calculation costs.

### 2.3. Finite Element Simulation

The calculation model was built using COMSOL software, COMSOL, Stockholm, Sweden. The thickness of the borophene material in the model was set to 1 mm, the diameter was set to 10 mm, and the upper and lower borophene are were layers. The refractive index of borophene material was from the first-principles calculation results. The optical parameters of air were from the COMSOL material library, and the real part of the refractive index was 1 and the imaginary part was 0. The simulation used the electromagnetic wave frequency domain module. The terahertz wave entered the air layer vertically from the upper boundary and then passed through the borophene layer. The lower boundary was the exit port of the terahertz wave, and the left and right boundaries adopted periodic boundary conditions. The grid division of the model adopted the physical field control grid. To improve the calculation efficiency, the air layer grid was coarsened, and to reduce the adverse effect of the material boundary layer on the calculation results, the boundary layer was refined to form a transition layer (as shown in Figure S2). The solver used a steady-state solver with a relative tolerance of 0.01.

The meshing method adopted in the finite element calculation was physical field control meshing. Generally, the physical field control mesh supported by COMSOL software can integrate calculation accuracy and calculation cost to yield the most optimized meshing, but for verifying the grid independence in the calculation process, the models of the 1-layer and 3-layer borophene structures at 1.4 THz (the intermediate frequency of 0.1 to 2.7 THz was selected as a representative) were also selected to test the grid independence. Four grid density gradients of 0.6, 0.8, 1.2, and 1.4 times the grid division density used in the paper were selected, respectively, and the reflectance and transmittance of the 1-layer and 3-layer borophene structures under the electromagnetic wave irradiation of 1.4 THz were calculated (the calculation results are shown in Table S1).

## 3. Results and Discussion

**Figure 1:**
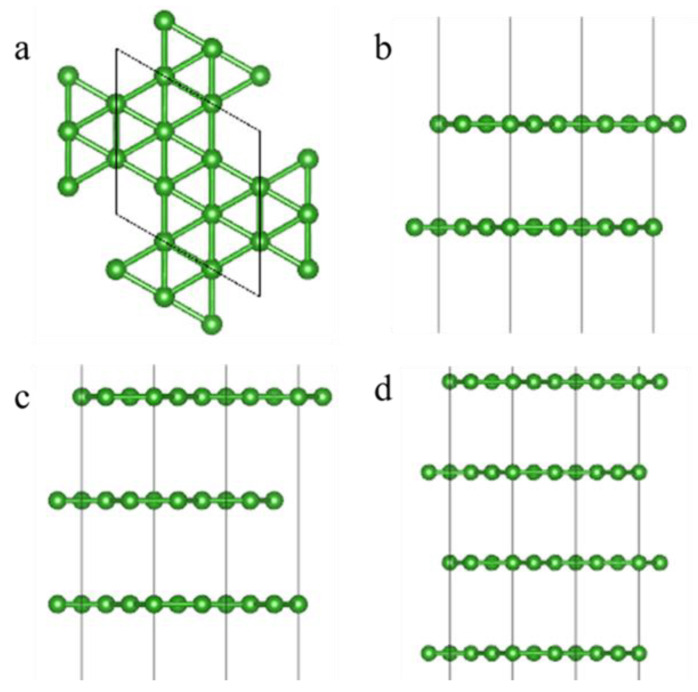
The structures of 1~4-layer α-sheet borophene. (**a**) Single-layer borophene combined with 8 atoms, (**b**) 2-layer borophene combined with 16 atoms, (**c**) 3-layer borophene combined with 24 atoms, and (**d**) 4-layer borophene combined with 32 atoms.

**Figure 2:**
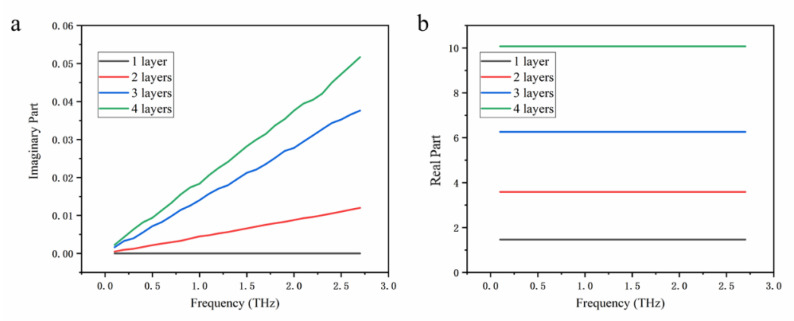
First-principles calculation of dielectric constants of borophene. (**a**) The imaginary part of the dielectric constant of 1~4-layer α-sheet structure varies with frequency. (**b**) The real part of the dielectric constant of 1~4-layer α-sheet structure varies with frequency.

First, the periodic molecular structures of single-layer and few-layer α-sheet borophene were established (the structures of 1~4-layer α-sheet borophene are shown in Figure 1). Then, structural optimization and static self-consistent calculations on the established structure were performed to obtain the wave function file of the structure, and they were used to calculate the real and imaginary parts of the dielectric constant of the single-layer and few-layer α-sheet borophene. As shown in Figure 2, the real and imaginary parts of the dielectric constant of the few-layer α-sheet borophene were obtained.

The above-mentioned dielectric constant that changes with the frequency is called the dynamic dielectric response function, which is recorded as ε(ω). After calculating the dynamic dielectric response function through the first principles, both the optics and spectra properties can be calculated. The specific calculation formula is shown in Equation (S1).

**Figure 3:**
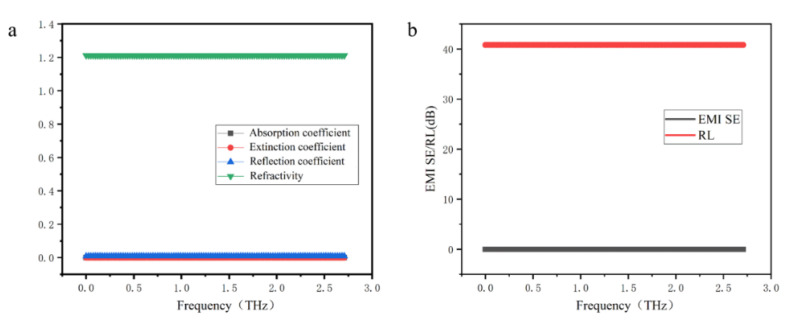
Optical properties and terahertz response performance of single-layer borophene. (**a**) Optical properties of single-layer borophene. (**b**) Terahertz response performance of single-layer borophene.

Since α-sheet borophene has a periodic lattice structure, to improve calculation efficiency, a single-layer α-sheet borophene composed of 8 boron atoms with a single-period lattice structure was established (Figure 1a). Based on this model, the main optical and spectra properties of single-layer α-sheet borophene were calculated, and the absorption coefficient, extinction coefficient, reflection coefficient, and refractive index of single-layer α-sheet borophene in the range of 0.1~2.7 THz were obtained (Figure 3a). The absorption coefficient and extinction coefficient of single-layer α-sheet borophene in the range of 0.1 to 2.7 THz were 0 within the calculation accuracy range (the two curves in the figure overlap at the ordinate 0). The reflection coefficient and refractive index did not change with frequency. In theory, the single-layer borophene has only one layer of boron atoms, which is almost equivalent to electromagnetic waves transparent material. When the terahertz beam is incident on its surface perpendicularly, most of the terahertz waves will pass through the single-layer borophene. Only a very small part of the electromagnetic waves will be reflected on the surface of the single-layer borophene or absorbed by boron atoms. Therefore, due to the calculation accuracy, the absorption coefficient and extinction coefficient were almost zero, and the reflection coefficient was also a small amount that did not change with frequency. According to the calculation method of transmittance and reflectance described in the Supplementary Materials, it can be seen that the transmittance of a single-layer borophene was 0.1 to 2.7 THz, which was approximately equal to 1 within the calculation accuracy. The reflectance was equal to the square of the reflection coefficient, which was 8.29 × 10^−5^, a very small value. The electromagnetic shielding effectiveness and reflection loss of a single-layer borophene calculated according to the transmission coefficient and reflection coefficient are shown in Figure 3. Since the transmittance was approximately equal to 1, the electromagnetic shielding effectiveness was approximately equal to 0, and the reflection loss was a constant of 40.8 dB (as in Figure 3b). It indicates that single-layer α-sheet borophene can be used as a low-reflectivity material for terahertz waves.

**Figure 4:**
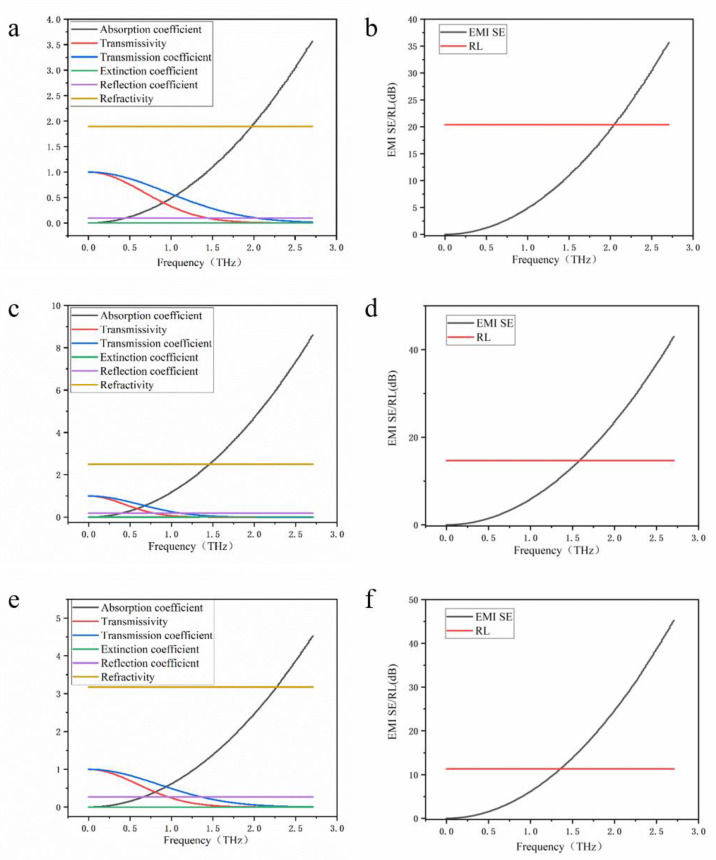
The terahertz response of few-layer borophene. (**a**) The optical properties of the two-layer structure, (**b**) terahertz response performance of the two-layer structure, (**c**) optical properties of the three-layer structure, (**d**) terahertz response performance of the three-layer structure, (**e**) optical properties of the four-layer structure, and (**f**) terahertz response performance of the four-layer structure.

Similar to studying the terahertz response performance of single-layer borophene, in order to improve the calculation efficiency, the smallest unit cells of 2-layer, 3-layer, and 4-layer α-sheet borophene were selected by 16, 24, and 32 atoms (Figure 1b–d). Using the same calculation method, the absorption coefficient, extinction coefficient, reflection coefficient, and refractive index of 2~4-layer α-sheet borophene in the range of 0.1~2.7 THz are shown in Figure 4. It is shown that the reflection coefficient and refractive index of borophene with 2 to 4 layers of α-sheet structure were similar to those of single-layer borophene, within the range of 0.1 to 2.7 THz, and neither changed with frequency. Unlike single-layer α-sheet borophene, the absorption coefficient of few-layer borophene increased with the frequency of the incident terahertz wave, which made the transmission of 2~4-layer α-sheet borophene decrease with the increase of the frequency of the terahertz wave. The negative logarithmic relationship between the transmission coefficient and the absorption coefficient was clearly expressed in the image (Figure 4a,c,e). In addition, since the transmission coefficient decreased with the increase of the terahertz wave frequency in the range of 0.1~2.7 THz, the electromagnetic shielding effectiveness (EMI SE) of the 2~4-layer α-sheet borophene calculated by the transmission coefficient varied with the frequency of the incident terahertz wave increases. The increasing trend of the frequency coincides with the increasing trend of the absorption coefficient. Since the reflection coefficient did not change with the increase of the frequency of the terahertz wave, the reflection loss (RL) of the 2~4-layer α-sheet borophene to the terahertz wave did not change with the change of the frequency (Figure 4b,d,f).

**Figure 5:**
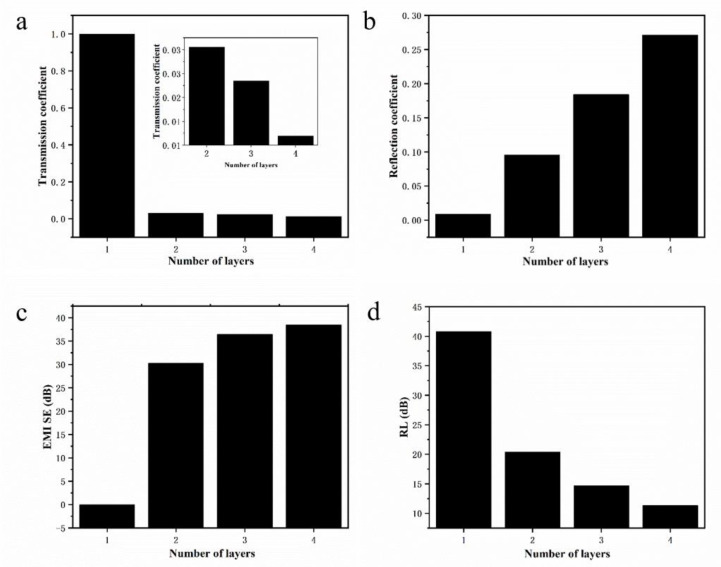
The influence of the number of layers on the terahertz response of borophene. (**a**) The change of transmission coefficient with the number of borophene layers, (**b**) the change of reflection coefficient with the number of borophene layers, (**c**) the change of electromagnetic shielding effectiveness with the number of borophene layers, and (**d**) the change of reflection loss with the number of borophene layers.

The calculation results indicate that there was a big difference in the response to terahertz waves between the few-layer α-sheet borophene and the single-layer borophene. The response performance to terahertz waves will also change as the number of borophene layers varied. To explore the effect of the number of layers on the terahertz response performance of the few-layer borophene, the different layers of the few-layer borophene at 2.5 THz were taken to conduct a lateral comparison study on the terahertz response characteristics. The bar graphs of the transmission coefficient, reflection coefficient, electromagnetic shielding effectiveness, and reflection loss as the number of layers increased are shown in Figure 5.

It was found that as the number of layers increased from two to four, the transmission coefficient decreased and the reflection coefficient increased. The inset in Figure 5a shows the change of the transmission coefficient of the borophene of two to four layers with the number of layers. It is shown that as the numbers of layers increased, the light transparency of the few-layer α-sheet borophene gradually decreased, and the absorption rate of terahertz waves increased. The increase in electromagnetic shielding effectiveness with the increase in the number of layers also illustrated this point. It is also worth noting that as the number of borophene layers increased from a single layer to four layers, its reflection loss (RL) continued to decrease. However, the decreasing trend gradually became smaller, and the value of reflection loss was always greater than 10 dB, which met the needs of terahertz stealth materials. In addition, as the number of layers increased, its maximum electromagnetic shielding effectiveness (at 2.7 THz) increased from 35.65 dB for two-layer borophene to 42.98 dB for three-layer borophene, and then to 45.24 dB for four-layer borophene. This is because the calculated result is the result after the normalization of the thickness, which is in an order of magnitude with the experimental result of the terahertz response of the few-layer borophene discussed later.

**Figure 6:**
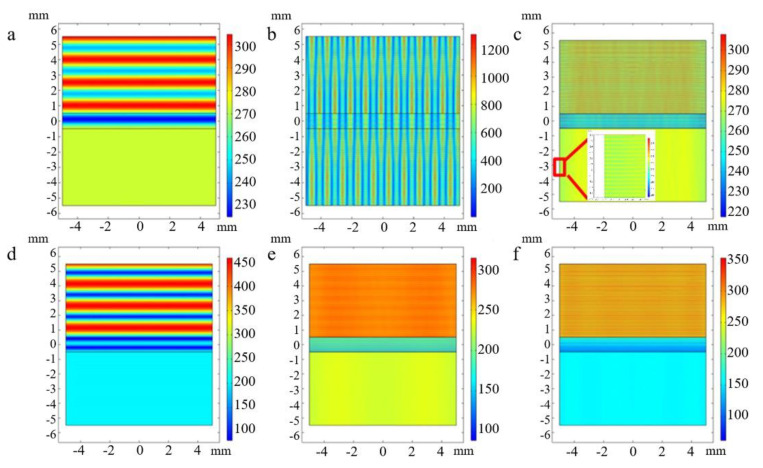
The electric intensity under terahertz waves. (**a**–**c**) The electric field intensity of single-layer borophene under the terahertz wave incident at 0.1, 1.5, and 2.7 THz. (**d**–**f**) The electric field strength of three-layer borophene under incident terahertz waves of 0.1, 1.5, and 2.7 THz.

The finite element simulation method was used to calculate the properties of the materials under different physical field conditions according to the different electromagnetic parameters of the materials. Single-layer borophene and three-layer borophene structures were selected as the research objects, and 0.1, 1.5, and 2.7 THz were selected as frequency variables for comparative analysis, as shown in Figure 6. Figure 6a–c show the electric field intensity changes of single-layer α-sheet borophene under 0.1, 1.5, and 2.7 THz waves. Figure 6d–f, respectively, show the change of electric field intensity of 3-layer α-sheet borophene under terahertz waves of 0.1, 1.5, and 2.7 THz waves. The results indicate that there are obvious interference fringes in the electric field intensity graph. This is because terahertz waves will be reflected when they are incident on the upper boundary of the borophene material from air. The terahertz waves will be reflected when reaching the lower boundary of the borophene material as well. The reflected wave will be further reflected many times at the upper and lower boundaries of the borophene material. The reflected wave with the incident wave, and the reflected wave with the reflected wave, have optical path differences, which will interfere with each other to form interference fringes.

In comparison, it was found that interference fringes also appeared in the lower part of the material for single-layer borophene when the frequency was greater than 0.1 THz. The reason is that it has optical transparency, and the absorption rate is very low for terahertz waves, so that part of the terahertz wave continues to propagate through the single-layer borophene material and interferes with the secondary reflected wave at the interface of the material. There were no interference fringes on the lower side of the material with the three-layer α-sheet borophene (discussed within the calculation accuracy), which indicates that the three-layer α-sheet borophene material has a very low transmittance to terahertz waves. The terahertz wave is reflected or absorbed when it is irradiated to the three-layer borophene, and the three-layer borophene has good terahertz shielding and stealth effects. The results are consistent with the above-mentioned first-principles simulation calculation results.

**Figure 7:**
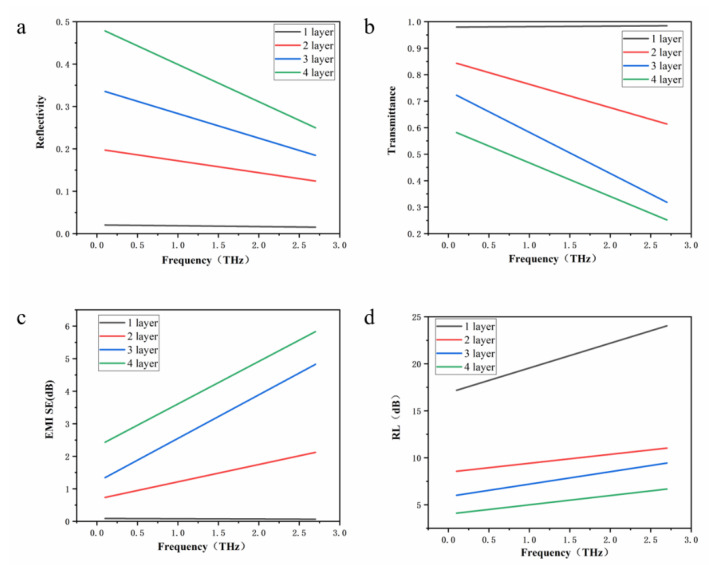
Terahertz response performance of borophene calculated by finite element: (**a**) 1–4-layer borophene reflectance change graph with frequency, (**b**) 1–4-layer borophene transmittance graph change with frequency, (**c**) EMI SE of 1~4-layer boropnene in the range of 0.1~2.7 THz, and (**d**) RL of 1~4-layer boropnene in the range of 0.1~2.7 THz.

The reflectance and transmittance of the 1–4-layer α-sheet borophene were output as shown in Figure 7. The reflectance and transmittance basically did not change with frequency for the single-layer α-sheet borophene. The reflectance value was close to 0 and the transmittance value was close to 1. It was also found that the absorptivity was almost 0. The electromagnetic shielding effectiveness was almost zero, which indicates that the single-layer borophene has terahertz wave transparency. The reflectivity and transmittance decreased with the increase of the incident terahertz wave frequency for 2~4-layer borophene. This indicates that the absorption rate of terahertz waves for 2~4-layer borophene increased with the increase of terahertz frequency in the range of 0.1~2.7 THz. Its electromagnetic shielding effectiveness increased with the increase of terahertz frequency, and with the increase of the number of borophene layers. The reflection loss changed very little with the frequency. The calculation results of the finite element simulation are basically consistent with the first-principles calculation results.

Since the prepared few-layer borophene samples were powder samples, to facilitate the study of the absorption and reflection properties of the few-layer borophene for terahertz waves in the terahertz time-domain spectroscopy system, the high terahertz wave transparency materials PDMS (polydimethylsiloxane) and high-resistance silicon were used as substrates for the preparation of terahertz performance test samples. The electromagnetic shielding effectiveness of PDMS and high-resistance silicon in the range of 0.1~2.7 THz is shown in Figure S1.

**Figure 8:**
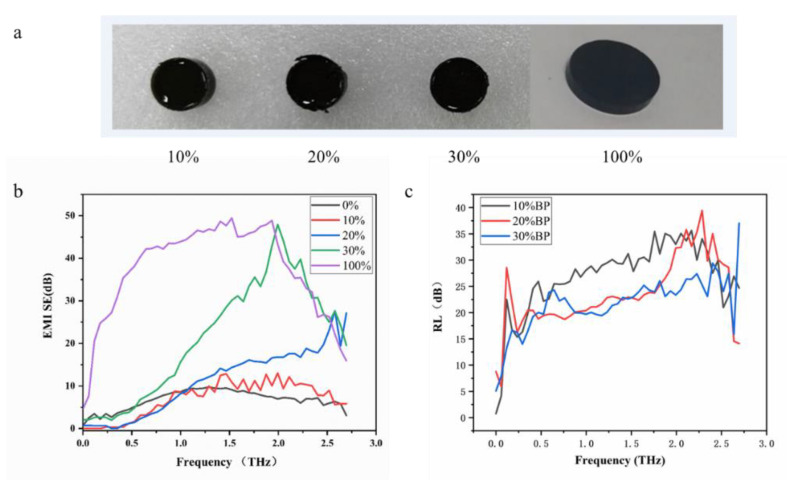
Terahertz response performance of composite materials with different borophene mixing concentrations. (**a**) PDMS composites with different mixing concentrations of few-layer borophene. (**b**) Influence of mixing concentration of few-layer borophene on the terahertz electromagnetic shielding effectiveness of PDMS composites. (**c**) Influence of mixing concentration of few-layer borophene on the terahertz reflection loss of PDMS composites.

Our research group have initially explored the terahertz response performance of few-layer borophene in previous papers and found that it has excellent terahertz stealth and shielding performance [16]. To clarify the influence of the mixing concentration of the few-layer borophene on the terahertz response performance of the PDMS composite material, we prepared the PDMS composite materials with 10%, 20%, and 30% mixing amounts of the few-layer borophene in the composite material. Few-layer borophene flakes made by pressing were prepared as well; that is, samples with 100% borophene content, as shown in Figure 8a. The transmission and reflection terahertz time-domain spectroscopy systems were used to test the transmission and reflection performance of the sample to the terahertz wave in the range of 0.1~2.7 THz. From Figure 8b, it can be concluded that the terahertz shielding effectiveness of the PDMS blank material without few-layer borophene mixing was less than 10 dB in the range of 0.1~2.7 THz. With the increase of the mixing concentration of the few-layer borophene, the terahertz electromagnetic shielding effectiveness for the PDMS composite material gradually improved. With the increase of the mixing concentration, the electromagnetic shielding effectiveness increased from 10 dB of the 10% mixing concentration to 50 dB of the 100% concentration (tablet) at the frequency of 2 THz.

On the other hand, it may be derived from different mixing concentrations of PDMS composite reflection losses in the range of 0.1~2.7 THz that the change of mixing concentration has little effect on terahertz reflective material loss (Figure 8c). The terahertz wave reflection loss of PDMS composite materials with different mixing concentrations was about 20 dB (0.1~2.7 THz), which meets the requirements of terahertz stealth materials for material reflection loss (>10 dB). Combining the above analysis of the electromagnetic shielding effectiveness of PDMS composites with different mixing concentrations, it can be concluded that: With the increase of the mixing concentration of few-layer borophene, the terahertz stealth and shielding effectiveness of PDMS composites will increase. In the specific application process, the mixing ratio of the few-layer borophene can be adjusted according to different scenarios to prepare the most suitable terahertz stealth and shielding materials.

Low-dimensional carbon materials such as graphene exhibit excellent performance in terahertz stealth and shielding. In the process of preparing a PDMS mixed few-layer borophene composite material, the different proportions of few-layer graphene were mixed and the terahertz time-domain spectroscopy system was used to explore the changes in the terahertz response performance of the composite material. The few-layer graphene used in the experiment was graphene nanosheets produced by Xianfeng Nano (outer diameter: 5~10 μm, thickness: 3~10 nm). Three mixing ratio samples (20% few-layer borophene, 10% few-layer borophene, and 10% few-layer graphene, 20% graphene) were prepared.

**Figure 9:**
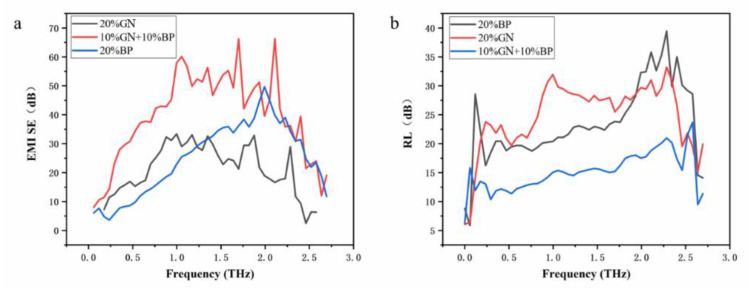
Terahertz response performance of graphene-mixed composites. (**a**) The influence of the terahertz electromagnetic shielding effectiveness. (**b**) The influence of the terahertz reflection loss.

The electromagnetic shielding effectiveness and reflection loss of the PDMS composites in the range of 0.1–2.7 THz with different mixing ratios of few-layer borophene and few-layer graphene obtained from the test are shown in Figure 9a. It can be found that the terahertz shielding efficiency of the PDMS composite material was the highest (up to 60 dB) when 10% few-layer borophene and 10% few-layer graphene were mixed. The terahertz shielding effectiveness of PDMS composites was relatively low (30–40 dB) with 20% few-layer borophene or 20% few-layer graphene mixed separately. The reason is maybe because a two-dimensional material network was formed inside the PDMS composite material when few-layer borophene or graphene was mixed. This network formed by the two-dimensional material is conducive to the absorption of terahertz waves. A more complex two-dimensional material network was formed inside the PDMS composite material when the few-layer borophene and the few-layer graphene were mixed at the same time, which increased the material’s absorption rate of terahertz waves and increased the electromagnetic shielding effectiveness. On the other hand, the PDMS composite material made by mixing with few-layer borophene and graphene at the same time had the smallest reflection loss (Figure 9b) compared with the single mixing of few-layer borophene or graphene (Figure 9b). It is worth mentioning that although the PDMS composite material made of both few-layer borophene and few-layer graphene had the smallest reflection loss, its reflection loss was still higher than 10 dB in the range of 0.1 to 2.7 THz. In summary, in the process of preparing a few-layer borophene-mixed PDMS composite material, the simultaneous mixing of few-layer graphene will help improve the terahertz stealth and shielding performance of the composite material.

## 4. Conclusions

In summary, we demonstrated for the first time that few-layer borophene has both superb terahertz shielding and stealth performance in the ultra-wide frequency range of 0.1~2.7 THz by theoretical and experimental methods. First-principles calculation results showed that in the range of 0.1~2.7 terahertz, single-layer borophene cannot be used as a terahertz stealth and shielding material due to its optical transparency. With the increase of the number of layers, the terahertz stealth and shielding performance of few-layer borophene will gradually increase. The EMI SE of 4-layer borophene reached 45.24 dB. In addition, the RL of 2~4-layer borophene was higher than 10 dB, which can be used as a terahertz stealth and shielding material. The finite element simulation calculation results were consistent with the first-principles calculation results. The experimental results of the PDMS composite material mixed with few-layer borophene under the terahertz time-domain spectroscopy system showed that the PDMS composite material mixed with few-layer borophene had good terahertz stealth and shielding performance. The EMI SE of the material can reach 50 dB, and the RL can reach 35 dB. It can be used as an excellent terahertz stealth and shielding material. With the increase of the mixing concentration of the few-layer borophene, the terahertz stealth and shielding performances of the composite material were gradually enhanced. In addition, the study also found that the composite material made by mixing few-layer borophene and graphene at the same time had better terahertz stealth and shielding performance than that mixed with few-layer borophene or graphene alone. In the actual application process, the mixing concentration and ratio of the material can be based on the working conditions. This study reveals the terahertz response performance of borophene for the first time, broadens the application range of borophene in terahertz stealth and shielding, and provides a reference for research on properties of borophene and terahertz stealth and shielding materials.

## Data Availability

Data are available via personal communication with proper reasons.

## Supplementary Materials

The following are available online at https://www.mdpi.com/article/10.3390/nano12152702/s1. Figure S1. The terahertz electromagnetic shielding effectiveness of the substrate. (a) EMI SE of PDMS in the range of 0.1~2.7 THz; (b) EMI SE of high resistance silicon in the range of 0.1~2.7 THz; Figure S2. Finite element simulation model. (a) Schematic diagram of the model; (b) Schematic diagram of meshing; Table S1. Verification of grid independence of finite element calculation.
