# Supplementary material for: Research and Application of Terahertz Response Mechanism of Few-Layer Borophene
Source: Nanomaterials (Basel). 2022 Aug 5;12(15):2702. doi: 10.3390/nano12152702 (PMC9370456; doi:10.3390/nano12152702)
Supplement: Supplementary file 1 [file nanomaterials-12-02702-s001.zip › nanomaterials-1834796-supplementary.pdf]

nanomaterials

MDPI

Supplementary Materials

Research and Application of Terahertz Response Mechanism of Few-Layer Borophene

Zhixun Zhang [1,†], Mingyang Yang [1,†], Yibo Zhang [1] and Ming Zhou [1,2,*]

1 State Key Laboratory of Tribology, School of Mechanical Engineering, Tsinghua University, Beijing 100084, China; qianyexunmengzzx@163.com (Z.Z.); yangmy@mail.tsinghua.edu.cn (M.Y.); zyb_tsinghua@163.com (Y.Z.)
2 Key Laboratory for Advanced Materials Processing Technology, Ministry of Education, Tsinghua University, Beijing 100084, China
* Correspondence: zhouming@tsinghua.edu.cn
† These authors contributed equally to this work.

Optical properties and spectral calculation formula:

$$R(\omega) = \left|\frac{\sqrt{\varepsilon(\omega)}-1}{\sqrt{\varepsilon(\omega)}+1}\right|^2 \quad (S1)$$

$$I(\omega) = (\sqrt{2})\omega\left[\sqrt{\varepsilon_1(\omega)^2 + \varepsilon_2(\omega)^2} - \varepsilon_1(\omega)\right]^{\frac{1}{2}} \quad (S2)$$

$$L(\omega) = \frac{\varepsilon_2(\omega)}{\varepsilon_1(\omega)^2+\varepsilon_2(\omega)^2} \quad (S3)$$

$$n(\omega) = (1/\sqrt{2})\omega\left[\sqrt{\varepsilon_1(\omega)^2 + \varepsilon_2(\omega)^2} + \varepsilon_1(\omega)\right]^{\frac{1}{2}} \quad (S4)$$

R (ω) is reflectivity, I (ω) is absorption coefficient, L (ω) is energy loss spectrum, n (ω) is refractive index and ε(ω) is dynamical dielectric response function. According to the absorption coefficient I (ω), the absorbance a of the material can be calculated, and then the transmittance t of the material to different THz bands can be obtained through the absorbance calculation. The calculation formula is as follows:

$$A = I(\omega)bc \quad (S5)$$

$$T = 10^{-A} \quad (S6)$$

Where *b* is the thickness of the material and *c* is the concentration of the material. Since it is a solid material, the concentration is taken as 1 mol/L. The electromagnetic shielding effectiveness (EMI SE) and reflection loss (RL) which characterize the stealth and shielding performance of the material can be calculated by the transmittance T and the reflection coefficient R obtained by simulation:

$$\text{EMI SE} = 20\log\frac{1}{T_E} \quad (S7)$$

$$RL = 20\log R_E \quad (S8)$$

T_E and R_E is the transmission coefficient and reflection coefficient of the material in different wave bands, separately. The transmittance and reflectivity are the square of the mode of transmission coefficient and reflection coefficient respectively.

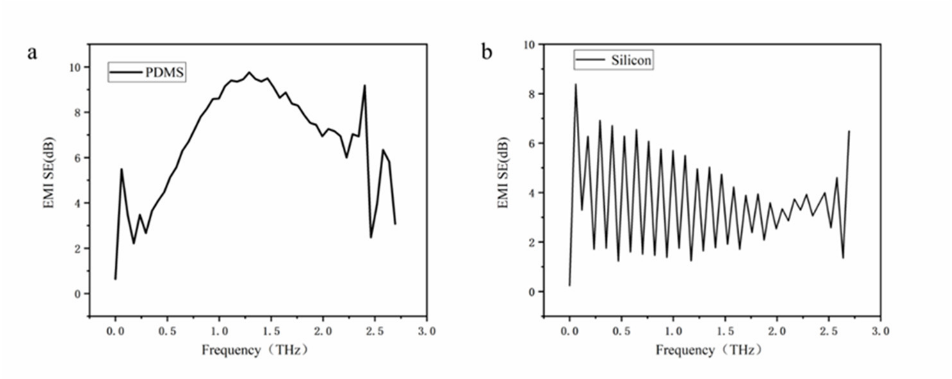

Figure S1. The terahertz electromagnetic shielding effectiveness of the substrate. (**a**) EMI SE of PDMS in the range of 0.1~2.7 THz; (**b**) EMI SE of high resistance silicon in the range of 0.1~2.7 THz.

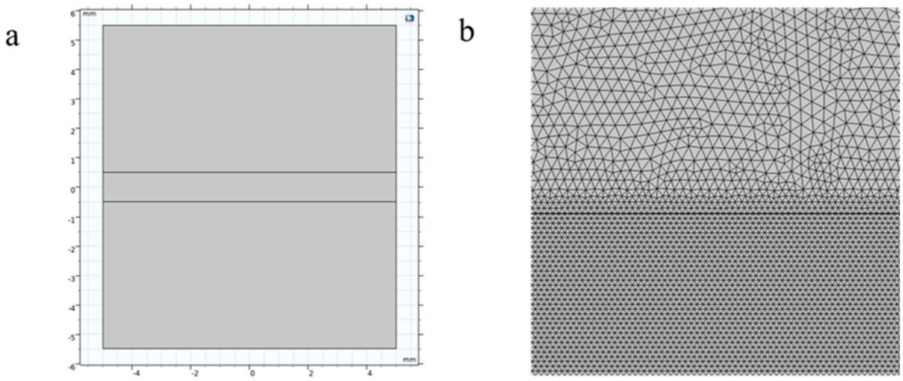

Figure S2. Finite element simulation model. (**a**) Schematic diagram of the model; (**b**) Schematic diagram of meshing.

Table S1. Verification of grid independence of finite element calculation.

Density Gradient			1-Layer		3-Layer	
	N_d	N_b	T	R	T	R
0.6	1738172	6174	0.0248	0.9752	0.3942	0.4441
0.8	2294780	7128	0.0248	0.9752	0.3921	0.4451
1.0	2995510	7970	0.0247	0.9753	0.3925	0.4453
1.2	3572108	8732	0.0248	0.9752	0.3944	0.4440
1.4	4138228	9428	0.0248	0.9752	0.3944	0.4438

N_d is the number of domain units; N_b is the number of boundary cells; T is the transmittance; R is the reflectivity.
